# Differing impact of the COVID-19 pandemic on youth mental health: combined population and clinical study

**DOI:** 10.1192/bjo.2023.601

**Published:** 2023-11-20

**Authors:** Lu Qi, Zuo Zhang, Lauren Robinson, Marina Bobou, Chantal Gourlan, Jeanne Winterer, Rebecca Adams, Kofoworola Agunbiade, Yuning Zhang, Sinead King, Nilakshi Vaidya, Eric Artiges, Tobias Banaschewski, Arun L. W. Bokde, M. John Broulidakis, Rüdiger Brühl, Herta Flor, Juliane H. Fröhner, Hugh Garavan, Antoine Grigis, Andreas Heinz, Sarah Hohmann, Marie-Laure Paillère Martinot, Sabina Millenet, Frauke Nees, Betteke Maria van Noort, Dimitri Papadopoulos Orfanos, Luise Poustka, Julia Sinclair, Michael N. Smolka, Robert Whelan, Argyris Stringaris, Henrik Walter, Jean-Luc Martinot, Gunter Schumann, Ulrike Schmidt, Sylvane Desrivières, IMAGEN Consortium, ESTRA Consortium and STRATIFY Consortium

**Affiliations:** Social, Genetic, and Developmental Psychiatry Centre, Institute of Psychiatry, Psychology and Neuroscience, King's College London, London, UK; Department of Psychological Medicine, Section for Eating Disorders, Institute of Psychiatry, Psychology and Neuroscience, King's College London, London, UK; and South London and Maudsley NHS Foundation Trust, London, UK; Institut National de la Santé et de la Recherche Médicale (INSERM) Research Unit 1299 ‘Trajectoires développementales en psychiatrie’, Université Paris-Saclay, Ecole Normale supérieure Paris-Saclay, CNRS, Centre Borelli, Gif-sur-Yvette, France; Department of Psychiatry and Psychotherapy, Charité – Universitätsmedizin Berlin, corporate member of Freie Universität Berlin, Humboldt-Universität zu Berlin, and Berlin Institute of Health, Campus Charité Mitte, Berlin, Germany; and Department of Education and Psychology, Freie Universität Berlin, Berlin, Germany; Psychology Department, University of Southampton, Southampton, UK; Social, Genetic, and Developmental Psychiatry Centre, Institute of Psychiatry, Psychology and Neuroscience, King's College London, London, UK; and School of Medicine, Center for Neuroimaging, Cognition and Genomics, National University of Ireland, Galway, Ireland; Centre for Population Neuroscience and Stratified Medicine, Department of Psychiatry and Neuroscience, Charité Universitätsmedizin Berlin, Berlin, Germany; Institut National de la Santé et de la Recherche Médicale Research Unit 1299 ‘Trajectoires développementales en psychiatrie’, Ecole Normale Supérieure Paris-Saclay, Université Paris-Saclay, CentreNational de la Recherche Scientifique 9010, Centre Borelli, Gif-sur-Yvette, France; and Department of Psychiatry, Etablissement Public de Santé Barthélemy Durand, Etampes, France; Department of Child and Adolescent Psychiatry and Psychotherapy, Central Institute of Mental Health, Medical Faculty Mannheim, Heidelberg University, Mannheim, Germany; Discipline of Psychiatry, School of Medicine and Trinity College Institute of Neuroscience, Trinity College Dublin, Dublin, Ireland; Clinical and Experimental Sciences, Faculty of Medicine, University of Southampton, Southampton, UK; and Department of Psychology, College of Science, Northeastern University, Boston, USA; Physikalisch-Technische Bundesanstalt, Braunschweig, Berlin, Germany; Institute of Cognitive and Clinical Neuroscience, Central Institute of Mental Health, Medical Faculty Mannheim, Heidelberg University, Mannheim, Germany; and Department of Psychology, School of Social Sciences, University of Mannheim, Mannheim, Germany; Department of Psychiatry and Neuroimaging Center, Technische Universität Dresden, Dresden, Germany; Departments of Psychiatry and Psychology, University of Vermont, Burlington, Vermont, USA; NeuroSpin, CEA, Université Paris-Saclay, Gif-sur-Yvette, France; Department of Psychiatry and Psychotherapy CCM, Charité – Universitätsmedizin Berlin, corporate member of Freie Universität Berlin, Humboldt-Universität zu Berlin, and Berlin Institute of Health, Berlin, Germany; Institut National de la Santé et de la Recherche Médicale Research Unit 1299 ‘Trajectoires développementales en psychiatrie’, Université Paris-Saclay, Ecole Normale supérieure Paris-Saclay, CNRS, Centre Borelli, Gif-sur-Yvette, France; and Department of Child and Adolescent Psychiatry, Pitié-Salpêtrière Hospital, AP-HP, Sorbonne University, Paris, France; Department of Child and Adolescent Psychiatry and Psychotherapy, Central Institute of Mental Health, Medical Faculty Mannheim, Heidelberg University, Mannheim, Germany; Institute of Cognitive and Clinical Neuroscience, Central Institute of Mental Health, Medical Faculty Mannheim, Heidelberg University, Mannheim, Germany; and Institute of Medical Psychology and Medical Sociology, University Medical Center Schleswig-Holstein, Kiel University, Kiel, Germany; Department of Psychology, MSB Medical School Berlin, Berlin, Germany; Dimitri Papadopoulos Orfanos, NeuroSpin, CEA, Université Paris-Saclay, Gif-sur-Yvette, France; Department of Child and Adolescent Psychiatry and Psychotherapy, University Medical Centre Göttingen, Göttingen, Germany; Clinical and Experimental Sciences, Faculty of Medicine, University of Southampton, Southampton, UK; School of Psychology and Global Brain Health Institute, Trinity College Dublin, Dublin, Ireland; Division of Psychiatry and Department of Clinical, Educational & Health Psychology, University College London, London, UK; Department of Psychiatry and Psychotherapy, Charité – Universitätsmedizin Berlin, corporate member of Freie Universität Berlin, Humboldt-Universität zu Berlin, and Berlin Institute of Health, Campus Charité Mitte, Berlin, Germany; Institut National de la Santé et de la Recherche Médicale Research Unit 1299 ‘Trajectoires développementales en psychiatrie’, Université Paris-Saclay, Ecole Normale supérieure Paris-Saclay, CNRS, Centre Borelli, Gif-sur-Yvette, France; Centre for Population Neuroscience and Stratified Medicine, Department of Psychiatry and Neuroscience, Charité Universitätsmedizin Berlin, Berlin, Germany; and Institut National de la Santé et de la Recherche Médicale Research Unit 1299 ‘Trajectoires développementales en psychiatrie’, Université Paris-Saclay, Ecole Normale supérieure Paris-Saclay, CNRS, Centre Borelli, Gif-sur-Yvette, France

**Keywords:** COVID-19, adolescent, depression, eating disorders, alcohol use disorder

## Abstract

**Background:**

Identifying youths most at risk to COVID-19-related mental illness is essential for the development of effective targeted interventions.

**Aims:**

To compare trajectories of mental health throughout the pandemic in youth with and without prior mental illness and identify those most at risk of COVID-19-related mental illness.

**Method:**

Data were collected from individuals aged 18–26 years (*N* = 669) from two existing cohorts: IMAGEN, a population-based cohort; and ESTRA/STRATIFY, clinical cohorts of individuals with pre-existing diagnoses of mental disorders. Repeated COVID-19 surveys and standardised mental health assessments were used to compare trajectories of mental health symptoms from before the pandemic through to the second lockdown.

**Results:**

Mental health trajectories differed significantly between cohorts. In the population cohort, depression and eating disorder symptoms increased by 33.9% (95% CI 31.78–36.57) and 15.6% (95% CI 15.39–15.68) during the pandemic, respectively. By contrast, these remained high over time in the clinical cohort. Conversely, trajectories of alcohol misuse were similar in both cohorts, decreasing continuously (a 15.2% decrease) during the pandemic. Pre-pandemic symptom severity predicted the observed mental health trajectories in the population cohort. Surprisingly, being relatively healthy predicted increases in depression and eating disorder symptoms and in body mass index. By contrast, those initially at higher risk for depression or eating disorders reported a lasting decrease.

**Conclusions:**

Healthier young people may be at greater risk of developing depressive or eating disorder symptoms during the COVID-19 pandemic. Targeted mental health interventions considering prior diagnostic risk may be warranted to help young people cope with the challenges of psychosocial stress and reduce the associated healthcare burden.

COVID-19 had detrimental effects on mental health, with worldwide rates of major depressive disorders (MDD) and anxiety disorders rising to 27.5 and 25.6%, respectively.^1^ Fear of the virus itself and lockdowns implemented by governments around the globe have caused greater mental distress and lower quality of life in the general population.^2–5^ In particularly, young people, who are known to experience major social role transitions,^6^ experienced higher levels of depressive and anxiety symptoms than people in older age groups.^1,4,5^ The pandemic has also been reported to worsen symptoms of patients with pre-existing mental illness,^7,8^ although contradictory findings have been reported.^9–12^ These contradictions and the limitations of studies to date highlight the need for further research that is both longitudinal and focuses on youth.^13^

The psychosocial stress caused by this pandemic has been detrimental to youth around the world, who have experienced adverse lifestyle changes.^14,15^ Confinement measures during lockdowns and the associated personal, educational and economic disruptions created pervasive social isolation, increased stress and decreased peer interactions, which may have triggered psychological distress and mental health difficulties in this age group. Indeed, meta-analyses of studies of children and adolescents indicate an increased prevalence of clinically elevated depression and anxiety symptoms compared with pre-pandemic estimates, especially in adolescent females.^16,17^ However, most studies investigated the effects of the pandemic on mental health changes only at the beginning of the pandemic. Although enormously instructive, these studies do not address the longer-term effects of the pandemic. Other limitations include the considerable heterogeneity of studies, which is largely due to differences in assessments and diagnostic criteria.^18^ The focus of most studies on anxiety and depression has also led to a call for more research to consider the effects of the pandemic on other youth mental health conditions that may have been negatively affected by the COVID-19 pandemic, in particular, eating disorders and addiction.^19^ More limited evidence available suggests that pre-pandemic disordered eating is a risk factor for poorer mental health during the pandemic.^8,20^ However, interpretations of these findings are limited as, again, assessment of mental health was restricted to the period of eased restrictions following the first lockdown. As for addiction, a decline in substance use has been reported, especially among adolescents initially at higher risk for substance use disorder.^21,22^ It is clear from these limitations that longitudinal trajectory research with comprehensive mental health assessments, spanning the pre-pandemic period and across multiple lockdown and release phases, is needed to understand the long-term impact of the COVID-19 pandemic on youth mental health.^13^ Research comparing data from the general population and from patient groups is also needed. Crucially, identifying the most vulnerable and resilient groups will be important for the design and delivery of the most appropriate targeted interventions.

## Aims

Our study addresses these needs by using data collected before and throughout the COVID-19 pandemic in two pre-existing youth cohorts: IMAGEN, a longitudinal population-based adolescent cohort; and ESTRA/STRATIFY, a clinical cohort with diagnoses of MDD, alcohol use disorders (AUD) and eating disorders. Our repeated assessments, based on the CoRonavIruS Health Impact Survey (CRISIS)^23^ and standardised mental health questionnaires, aimed to (i) establish trajectories of behaviours and mental health symptoms throughout stages of the pandemic in these cohorts; (ii) compare these trajectories to identify the most vulnerable groups; and (iii) identify pre-pandemic predictors of these mental health trajectories.

## Method

### Study design

Participants were drawn from three existing cohorts located in the UK, France and Germany: IMAGEN, STRATIFY and ESTRA. IMAGEN was a longitudinal population cohort, whereas STRATIFY and ESTRA were case–control cohorts. To be eligible for inclusion, participants needed to respond to our invitation and provide informed consent through an online form sent via email. Data collection was conducted through online questionnaires, with the initial round taking place during the first national lockdown in the UK and Europe (April–May 2020). Subsequent follow-up surveys were administered when the first lockdown was released (July 2020) and when the second lockdown was imposed (November 2020). The design and reporting of our study were in accordance with the STROBE (Strengthening the Reporting of Observational Studies in Epidemiology) guidelines.

### Participants

#### Population cohort

These participants, with no known history of mental illness, were drawn from the IMAGEN study, a longitudinal cohort of over 2000 adolescents recruited at age 14 years from eight study sites in Europe, with follow-up assessments at ages 16, 19 and 23 years. For detailed study protocols, please refer to Schumann et al.^24^ Our survey was sent to those who had completed the follow-up assessment at age 23 (*N* = 1350). A total of 458 IMAGEN participants recruited from the UK, France and Germany (London, Nottingham, Paris, Mannheim and Berlin) who completed the COVID-19 survey at baseline were included in our analyses.

#### Clinical cohort

This cohort was derived from two studies, STRATIFY and ESTRA, of participants aged 18–30 years (*N* = 628). STRATIFY participants included in this study comprised participants recruited in the UK and Germany (London, Southampton and Berlin) who met diagnostic criteria for MDD and AUD, as assessed by self-report via online computerised screening. Participants were included if they had scores ≥15 (moderate to severe) on the Patient Health Questionnaire (PHQ-9)^25^ and Alcohol Use Disorders Identification Test (AUDIT)^26^ for MDD and AUD, respectively. ESTRA consisted of participants recruited in London and meeting the DSM-5^27^ diagnostic criteria for anorexia nervosa or bulimia nervosa. All were female. Their eating disorder symptoms were assessed using the Eating Disorder Diagnostic Scale (DSM-5 version) over a screening phone call by study researchers.^28^ A total of 211 STRATIFY/ESTRA participants (80 MDD, 51 AUD, 47 anorexia nervosa and 33 bulimia nervosa) who completed the COVID-19 survey at baseline were included in our analyses.

### Ethics statement

The authors assert that all procedures contributing to this work comply with the ethical standards of the relevant national and institutional committees on human experimentation and with the Helsinki Declaration of 1975, as revised in 2008. All procedures involving human subjects/patients were approved by King's College London Research Ethics Committee (17/LO/0552) for IMAGEN, London Westminster Research Ethics Committee (PNM/10/11-126) for STRATIFY and North West–Greater Manchester South Research Ethics Committee (20/NW/0143) for ESTRA. All adult participants provided written/online signature informed consent to participate in this study.

### Survey and assessments

#### The COVID-19 survey

We adapted the CoRonavIruS Health Impact Survey (CRISIS v0.1 http://www.crisissurvey.org)^23^ to examine changes to individuals’ mental health and behaviours induced by the pandemic. The survey encompassed data collection at various time points, specifically: pre-pandemic (3 months prior, pre-LD1), during the first lockdown (LD1), after the first lockdown (after-LD1) and during the second lockdown (LD2). This questionnaire assessed a range of data domains including COVID-19-related health status and life changes, daily behaviours and emotions, and worries due to the COVID-19 crisis (see Supplementary Information available at https://doi.org/10.1192/bjo.2023.601 for details).

#### Mental health assessments

The severity of mental disorder symptoms was assessed with validated questionnaires, including the PHQ-9 for depressive symptoms, the Eating Disorder Examination Questionnaire (EDE-Q)^29^ for eating disorder symptoms and the AUDIT Consumption^26^ for alcohol misuse (see Supplementary Methods for details). Questionnaires were administered at three time points: (a) at the previous recruitment wave, ~3 years prior to the pandemic (pre-PD), (b) during the first lockdown (LD1) and (c) during the second lockdown (LD2). The exception was the EDE-Q, which was administered at only two time points (i.e. LD1 and LD2) in the clinical cohort.

#### Pre-pandemic mental health

Pre-PD symptom severity scores were used to classify participants from the population cohort, based on the following criteria. For depression, PHQ-9 scores of 0–4, 5–9 and 10+ were used to indicate minimal, mild and moderate to severe depression, respectively.^25^ For alcohol misuse, AUDIT scores of 0–7 and 8+ were used to indicate low and high risk, respectively.^30^ For eating disorders, EDE-Q global scores <2.8 (for females) or 1.68 (for males) were used to indicate low risk; higher scores were considered to indicate probable eating disorders.^31,32^ For body mass index (BMI), we used the following categories: underweight or normal weight, BMI < 25; overweight or obese: BMI > 25.

### Statistical analyses

Data were analysed in SPSS version 27 using mixed-effects analysis of variance (ANOVA), with within-subject effect (time) adjusted by country and between-subjects effects (cohort and sex) adjusted by country and age. For each analysis, participants were included if they had no missing data for any variable needed. Separate analyses were conducted, as detailed in the Supplementary Methods, on the whole sample or on each cohort separately. Statistical significance was set at *P* < 0.05.

#### Trajectories of lifestyle changes, worries and mental health symptoms during the pandemic

Scores from the COVID survey and mental health questionnaires were analysed across time points. In addition to time effects, we investigated cohort and sex effects, along with interaction effects (i.e. time × cohort, time × sex) in the whole sample. Given the strong cohort effects, we also investigated these trajectories in the population and clinical cohorts separately.

#### Trajectories of mental health symptoms based on pre-pandemic symptom severity

These analyses were performed in the population cohort only. Subgroups based on the severity of pre-PD symptoms (see above) were included in mixed-effects ANOVAs. Three analyses were run to investigate interactions between time and pre-PD severity of mental health symptoms (i.e. depression, alcohol misuse or eating disorder) during the pandemic.

## Results

### Sample description and participants’ characteristics

**Fig. 1:**
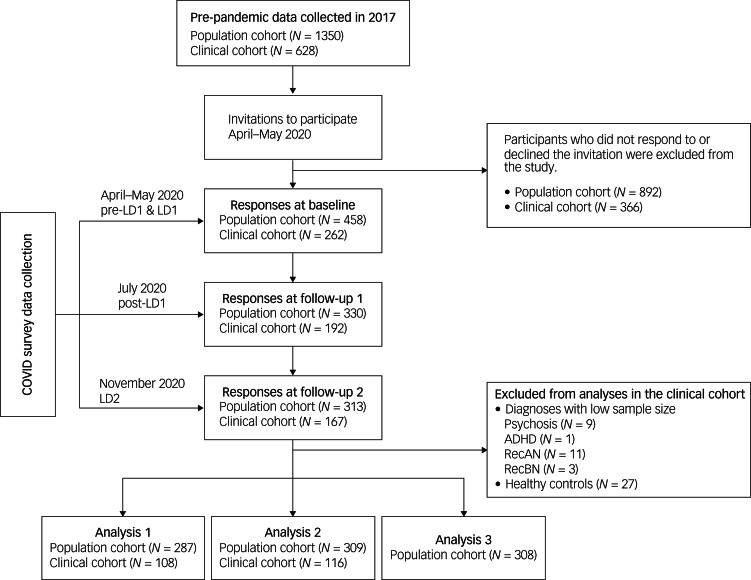
Recruitment flowchart for study participants. Analysis 1 examined trajectories of behaviours, emotions and COVID-related worries during the pandemic. Analysis 2 investigated mental health trajectories during the pandemic. Analysis 3 explored the impact of pre-pandemic symptom severity on mental health trajectories during the pandemic. For each analysis, participants were excluded if they had missing data for any required variable. ADHD, attention-deficit hyperactivity disorder; LD1, first lockdown; LD2, second lockdown; RecAN, recovered from anorexia nervosa; RecBN, recovered from bulimia nervosa.

A flowchart outlining the recruitment and follow-up of participants for this study is provided in Fig. 1. In total, 669 individuals (31.5% clinical cohort; 69.4% females) completed the COVID survey at pre-LD1, 471 (29.9% clinical cohort) at LD1 and 429 (27.0% clinical cohort) at LD2 (Supplementary Table 1). As expected, immediately prior to the pandemic, symptoms of depression (F(1,615) = 156.26, *P* < 0.001, η_p_^2^ = 0.203) and alcohol misuse (F(1,630) = 30.11, *P* < 0.001, η_p_^2^ = 0.046) were higher in the clinical cohort. BMI was higher in the population sample (F(1,543) = 17.76, *P* < 0.001, η_p_^2^ = 0.032). Females reported higher levels of depressive (F(1,615) = 13.95, *P* < 0.001, η_p_^2^ = 0.022) and eating disorder symptoms (F(1,411) = 45.92, *P* < 0.001, η_p_^2^ = 0.100), whereas males reported higher levels of alcohol misuse (F(1,630) = 23.38, *P* < 0.001, η_p_^2^ = 0.036).

### Behavioural, emotional and mental health trajectories during the pandemic

**Fig. 2:**
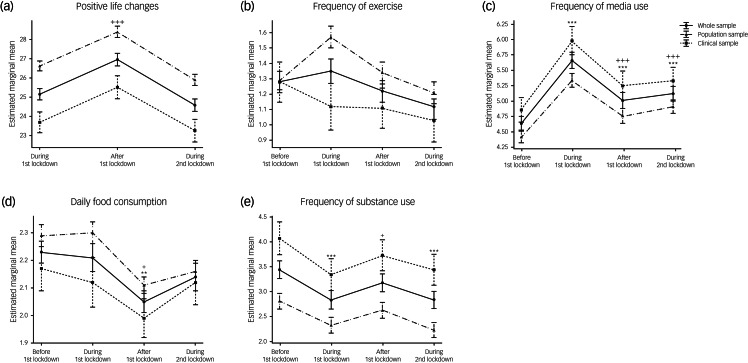
Behavioural trajectories during the pandemic, including (a) positive life changes; (b) frequency of exercising; (c) frequency of media use; (d) daily food consumption; and (e) frequency of substance use, in the whole sample and stratified by cohort. Data are expressed as mean and standard error. Time effects from mixed-effects ANOVA in the whole sample were estimated by comparing data collected before the first lockdown with data collected at other time points (*P < 0.05, **P < 0.01, ***P < 0.001) and by comparing data collected during the first lockdown with data collected afterwards (^+^*P* < 0.05, ^++^*P* < 0.01, ^+++^*P* < 0.001).

**Fig. 3:**
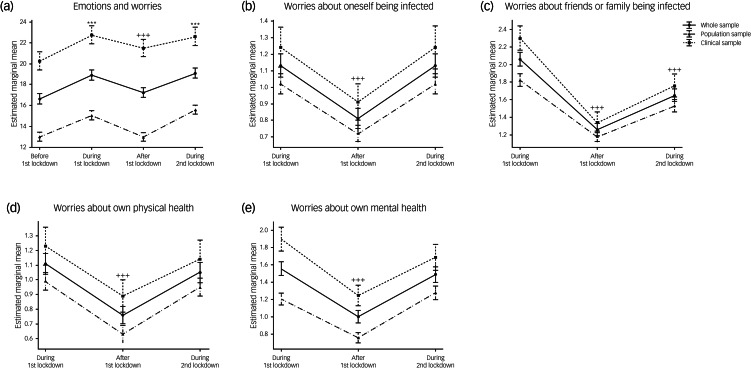
Emotional trajectories during the pandemic, including (a) emotions and worries; (b) worries about oneself being infected; (c) worries about friends or family being infected; (d) worries about own physical health; and (e) worries about own mental health, in the whole sample and stratified by cohort. Data are expressed as mean and standard error. Time effects from mixed-effects ANOVA in the whole sample were estimated by comparing data collected before the first lockdown with data collected at other time points (*P < 0.05, **P < 0.01, ***P < 0.001) and by comparing data collected during the first lockdown with data collected afterwards (^+^*P* < 0.05, ^++^*P* < 0.01, ^+++^*P* < 0.001).

We compared behavioural, emotional and mental health trajectories during the pandemic in our two cohorts using mixed-effects ANOVA. Significant main effects of time on behaviours (i.e. positive lifestyle changes, frequency of media use, average daily food consumption and frequency of substance use; all *P* < 0.001; Fig. 2(a–e) and Supplementary Table 2) were observed when analysing both samples together, but there were no significant time × cohort interactions. Similarly, there were significant main effects of time on emotional health, as assessed by the ‘emotions and worries’ and ‘worries about COVID’ sections of the survey (all *P* < 0.001; Fig. 3(a–e) and Supplementary Table 2) but no significant time × cohort interactions (all detailed in the Supplementary Material).

**Fig. 4:**
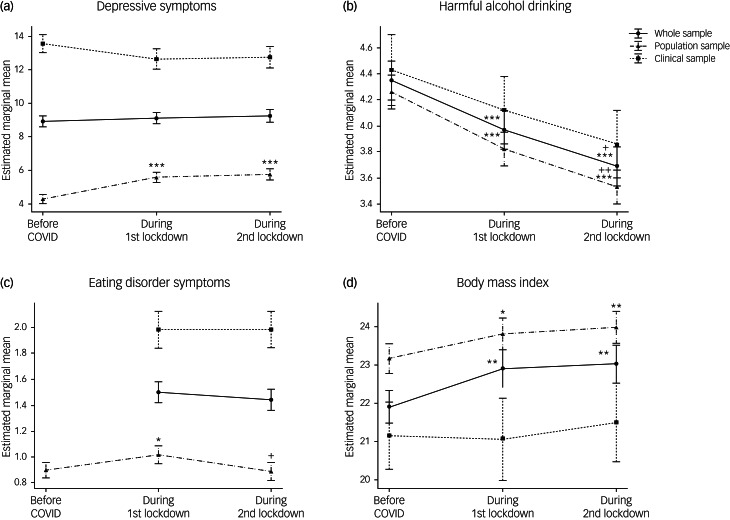
Mental health trajectories during the pandemic. Trajectories of (a) depressive symptoms; (b) harmful alcohol drinking; (c) eating disorder symptoms (d) and body mass index are indicated for the whole sample and for each cohort separately. Data are expressed as mean and standard error. Time effects from mixed-effects ANOVA in the whole sample were estimated by comparing data collected before the pandemic with data collected at other time points (*P < 0.05, **P < 0.01, ***P < 0.001), and by comparing data collected during the first lockdown with data collected afterwards (^+^*P* < 0.05, ^++^*P* < 0.01, ^+++^*P* < 0.001).

Comparisons of mental health symptoms (i.e. depression, alcohol drinking and eating disorders) and BMI just prior to the pandemic and during both lockdowns revealed the differential impact of the COVID-19 crisis on the cohorts (Fig. 4(a–d) and Supplementary Table 3).

There was no significant main effect of time on depressive symptoms (F(2,836) = 0.43, *P* = 0.65, η_p_^2^ = 0.001) in the whole sample. As expected, there were sex (F(1,417) = 11.84, *P* < 0.001, η_p_^2^ = 0.028) and large cohort effects (F(1, 1417) = 213.05, *P* < 0.001, η_p_^2^ = 0.338), depressive symptoms being higher in females and in the clinical cohort. A significant time × cohort interaction was also found (F(2,836) = 7.41, *P* < 0.001, η_p_^2^ = 0.017), indicating that the trajectories of depressive symptoms significantly differed between the population and the clinical samples (Fig. 4(a)). Analyses of these trajectories in the two cohorts separately revealed a significant main effect of time only in the population cohort (F(2,606) = 16.98, *P* < 0.001, η_p_^2^ = 0.053). Depressive symptoms increased by 33.9% (95% CI, 31.78–36.57) during the lockdowns, with severity increasing to mild depression compared with minimal depression prior to the pandemic. In the clinical cohort, depressive symptoms remained high and constant across time (F(2,220) = 0.10, *P* = 0.91, η_p_^2^ = 0.001).

A significant main effect of time in harmful alcohol drinking was found (F(2,838) = 14.06, *P* < 0.001, η_p_^2^ = 0.032), with symptoms decreasing during the pandemic to reach their lowest levels (i.e. a 15.2% decrease) during the second lockdown (pre-PD > LD1 > LD2; *P* < 0.05). Males drank more than females (F(1,418) = 41.90, *P* < 0.001, η_p_^2^ = 0.091). There were no significant cohort or time × cohort interactions (Fig. 4(b)). Nonetheless, a time × diagnosis interaction in the clinical cohort (F(6,220) = 4.25, *P* < 0.001, η_p_^2^ = 0.104) revealed that the significant the decline in harmful drinking in the clinical cohort (i.e. a 23.04% decrease) was driven by participants with AUD.

For eating disorder symptoms, as the EDE-Q was only administered at two time points in the clinical cohort, we analysed the two cohorts separately. In the population cohort, there was a significant main effect of time on eating disorder behaviours and attitudes, as assessed by the EDE-Q global score (F(2,550) = 4.31, *P* = 0.01, η_p_^2^ = 0.015). Eating disorder symptoms increased by 15.6% (95% CI, 15.39–15.68) during the first lockdown, returning to pre-pandemic levels during the second lockdown (Fig. 4(b)). As expected, eating disorder symptoms were significantly higher in females than males (F(1, 274) = 33.26, *P* = <0.001, η_p_^2^ = 0.108), but there were no significant time × sex interactions (F(2, 550) = 1.14, *P* = 0.32, η_p_^2^ = 0.004). In contrast to our findings in the population cohort, eating disorder symptoms did not significantly differ between the two lockdowns in the clinical cohort (F(1,110) = 0.09, *P* = 0.77, η_p_^2^ = 0.001). Limiting analyses to the eating disorder subgroups also revealed no significant time effects (F(1,30) = 2.35, *P* = 0.14, η_p_^2^ = 0.073 and F(1,18) = 0.03, *P* = 0.87, η_p_^2^ = 0.002), for anorexia nervosa and bulimia nervosa, respectively.

Analyses of BMI trajectories revealed a significant main effect of time in the whole sample (F(2, 638) = 6.85, *P* < 0.001, η_p_^2^ = 0.032), with higher BMIs during the pandemic (pre-PD < LD1 and LD2, *P* < 0.01) (Fig. 4(d)). A significant time × sex interaction (F(2,638) = 3.81, *P* < 0.05, η_p_^2^ = 0.012) indicated that BMI significantly increased in females but not in males. No time × cohort interaction was found, but analyses of the two cohorts separately indicated that these findings were driven by the population cohort (F(2,428) = 9.61, *P* < 0.01, η_p_^2^ = 0.043). Although no main effect of time was found in the clinical cohort, analyses within each diagnostic group revealed a time × sex interaction (F(2,50) = 5.48, *P* < 0.01, η_p_^2^ = 0.180) in the AUD group, with significant BMI increases observed only in females (pre-PD < LD2, *P* < 0.05).

Re-running the analyses described above while controlling for other potential confounders generated largely similar results (Supplementary Table 3).

### Effects of pre-pandemic symptom severity on mental health trajectories during the pandemic

**Fig. 5:**
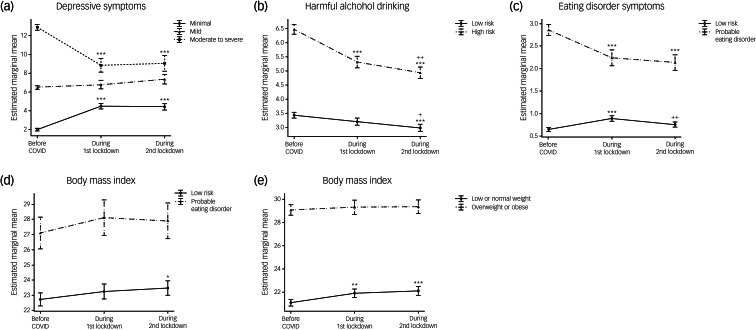
Effects of pre-pandemic symptom severity on pandemic-induced mental health trajectories. (a) Effects of pre-pandemic severity for depression (minimal, mild and moderate to severe) on trajectories of depressive symptoms; (b) effects of pre-pandemic risk for alcohol misuse (low or high risk) on trajectories of harmful alcohol drinking; (c) effects of pre-pandemic risk for eating disorders (low risk or probable eating disorder) on trajectories of eating disorder symptoms; (d) effects of pre-pandemic risk for eating disorders on body mass index (BMI) trajectories, and (e) effects of pre-pandemic BMI (low or normal and overweight or obese) on BMI trajectories. Data are expressed as mean and standard error. Mixed-effects ANOVA revealed significant time × group (i.e. pre-pandemic risk levels) interactions in all comparisons. Time effects in each group were estimated by comparing data collected before the pandemic with data collected at other time points (*P < 0.05, **P < 0.01, ***P < 0.001) and by comparing data collected during the first lockdown with data collected afterwards (^+^*P* < 0.05, ^++^*P* < 0.01, ^+++^*P* < 0.001).

The following analyses were performed to identify participants from the population cohort most vulnerable to COVID-induced mental illness. We categorised participants from this cohort based on their pre-pandemic symptom severity with respect to depression, alcohol misuse, eating disorders and BMI and re-ran analyses with these categories as predictors (Fig. 5(a–e) and Supplementary Table 4).

#### Effects of pre-pandemic depression symptom severity on depressive symptom trajectories

There were significant interactions between time and pre-pandemic symptom severity for pandemic-related depressive symptoms (F(4,602) = 21.35, *P* < 0.001, η_p_^2^ = 0.124). *Post hoc* analyses revealed notable group differences in trajectories (Fig. 5(a) and Supplementary Table 4). Participants with minimal pre-pandemic depression symptoms reported significant changes over time (F(2,300) = 41.73, *P* = <0.001, η_p_^2^ = 0.218), with symptoms increasing during the first lockdown and remaining higher afterwards (pre-PD < LD1 or LD2, *P* < 0.001). By contrast, participants with moderate to severe depression reported the opposite trend (F(2,300) = 17.54, *P* = <0.001, η_p_^2^ = 0.105), with symptoms being lower during the first and second lockdowns (pre-PD > LD1 or LD2, *P* < 0.001). Participants with mild depression did not report significant symptom changes with time (F(2,300) = 1.59, *P* = 0.21, η_p_^2^ = 0.010).

#### Effects of pre-pandemic risk for alcohol misuse on trajectories of alcohol misuse

There were significant group differences in trajectories of harmful drinking during the pandemic (F(2,606) = 16.26; *P* = <0.001, η_p_^2^ = 0.051; Fig. 5(b) and Supplementary Table 4). Participants initially more at risk of harmful drinking (i.e. prior to the pandemic) reported a significant decrease in alcohol misuse at all time points during the pandemic (pre-PD > LD1 > LD2, all, *P* < 0.001). A decrease was also observed for participants at low risk, and this became significant during the second lockdown (pre-PD > LD2, *P* < 0.001; LD1 > LD2, *P* < 0.05).

#### Effects of pre-pandemic risk for eating disorder on eating disorder symptoms and BMI trajectories

Similarly, significant group differences in trajectories of eating disorder symptoms during the pandemic were observed (F(2, 548) = 18.07; *P* = <0.001, η_p_^2^ = 0.062; Fig. 5(c) and Supplementary Table 4). Participants initially at low risk for eating disorders reported a increase in eating disorder symptoms specifically during the first lockdown, with symptoms decreasing during the second lockdown (pre-PD < LD1, *P* = <0.001; LD1 > LD2, *P* < 0.01). Conversely, for participants initially scoring higher for eating disorder symptoms (i.e. those with probable eating disorder), symptoms significantly decreased during the first lockdown (*P* < 0.001), remaining lower during the second lockdown. Unsurprisingly, there were significant group differences in BMI (F(1,195) = 16.03; *P* < 0.001, η_p_^2^ = 0.076), with participants with probable eating disorder having BMIs in the overweight range and those at low risk having BMIs in the normal range (Fig. 5(d)). No significant group differences in BMI trajectories during the pandemic were observed (F(2,392) = 0.43; *P* = 0.65, η_p_^2^ = 0.002); a nominally significant increase in BMI was observed in the participants at low risk for eating disorder (pre-PD < LD2, *P* < 0.05) but not in those with higher eating disorder risk (Fig. 5(d)).

#### Effects of pre-pandemic BMI on BMI and eating disorder symptoms trajectories

Although no significant group differences on BMI trajectories were observed when comparing participants who were initially underweight/normal weight (BMI < 25) and overweight/obese (BMI > 25) (F(2,426) = 2.09; *P* = 0.13, η_p_^2^ = 0.010), significant increases in BMI were observed in the underweight/normal weight group (pre-PD < LD1, *P* = 0.005; pre-PD < LD2, *P* < 0.001) but not in the overweight/obese group, for which BMI remained constant during the pandemic (Fig. 5(e) and Supplementary Table 4). Consistent with the analyses above, the increase in BMI in the underweight/normal weight group was paralleled by a significant increase in eating disorder symptoms, specifically during the first lockdown (BMI < 25; F(2,270) = 5.74, *P* = 0.004, η_p_^2^ = 0.041; pre-PD < LD1, *P* = 0.003; LD1 > LD2, *P* = 0.045).

When re-running analyses controlling for other potential confounders, minor differences emerged in *post hoc* tests, probably owing to increased degrees of freedom and reduced sample size after adding numerous covariates. However, the overall pattern remained – participants with high levels of pre-pandemic symptoms showed improvement during lockdowns, whereas those with minimal pre-pandemic depression symptoms reported significant increases over time.

## Discussion

This comparative study following population and clinical cohorts during the pandemic revealed the differing impact of the pandemic in youth with and without pre-existing mental illness. Whereas symptoms of depression and eating disorders increased during the pandemic in young people from the population, these symptoms remained high and stable in the clinical cohort. Pre-pandemic symptom severity predicted mental health trajectories in the population cohort. Participants initially at higher risk for depression, alcohol misuse or eating disorders reported a lasting decrease in their symptoms over the course of the pandemic. By contrast, being relatively healthy (i.e. having the lowest scores for depression or eating disorder) was a significant risk for deterioration in mental health during the pandemic; this was associated with relative increases in depressive symptoms throughout the pandemic and in eating disorder symptoms during the first lockdown. Being non-overweight or non-obese predicted the observed rise in eating disorder symptoms and was associated with weight gain (i.e. BMI increase).

Our findings corroborate previous research showing an increase in depression symptoms in all age groups^5,15,16,33^ from the population during the pandemic, but particularly in young and more physically active individuals.^15^ This observation may reflect greater changes in lifestyle habits in this group or a reduced tolerance of uncertainty. Our findings also highlight the contrasting effects of the pandemic on other mental health outcomes in young people: a long-term negative impact on depressive symptoms lasting until the second lockdown, in contrast to the transient increase in eating disorder symptoms and continuous decrease in alcohol misuse.

Our findings also shed light on the contradictory debate concerning pre-existing mental illnesses.^7–12,34^ Contrary to previous reports of worsening symptoms during the pandemic in patients with a history of mental illness^7^ or pre-existing disordered eating,^8^ our findings indicated that although symptoms remained higher in the clinical sample, they did not worsen because of the pandemic. These discrepancies may be due to a lack of diagnostic measurement of mental illness and lack of repeated assessments to measure symptom changes during the pandemic in the relevant studies. Our observations of differences in mental health trajectories between young people from the general population and those with a clinical diagnosis suggest that pre-pandemic symptoms may have been a protective factor, and that the general population was more likely to be affected by the lockdowns than patients, which our analyses confirmed. The clinical cohort seemed to be resilient in the face of the pandemic, confirming previous reports for depression from the early stages of the pandemic^10,11,35^ and further indicating that this effect persisted as the pandemic progressed. By contrast, and in agreement with previous assessments of depression in adults^9^ and adolescents,^12^ young people without depressive or eating disorder symptoms showed an increase in these symptoms during the pandemic, whereas those with the highest pre-pandemic risk experienced a decrease. However, it should be noted that symptoms in the higher-risk groups remained much higher than those of individuals without prior symptoms, and that patients are more vulnerable to some stressful situations due to the pandemic.^34^

In contrast to our findings for depression and eating disorders, we observed a decline in alcohol and substance misuse during the pandemic, consistent with previous evidence.^22,36^ This decline during lockdown periods was similar in participants with and without mental health diagnoses. Among the general population, this decline could be attributed to both those at high risk and those at low risk for alcohol misuse and may reflect closures of shops, bars and pubs during lockdown.

Participants from the general population and patients differed in the intensity of their behavioural or emotional responses to the pandemic but not in their trajectories. That young people are not equally at risk from the psychosocial stress brought about by COVID-19 was to be expected; however, counterintuitively, our findings indicate that healthier individuals tended to be the most vulnerable to the negative effects of the pandemic on mental health, not those with a higher burden. Possible explanations for this are that heightened fears and worries during periods of confinement, as highlighted in this study, and increased social isolation may have contributed to deterioration of mental health in healthier individuals. By contrast, those with depression and eating disorders might have felt relief owing to reduced exposure to psychosocial stressors (e.g. social interactions). They may also have felt less isolated given the global increase in fears and worries. As for alcohol and substance use, as noted above, the general reduction may reflect restriction policies such as closures of shops, bars and pubs, which would have limited access to those substances, as evidenced by a return to pre-pandemic levels after confinement measures were lifted. In addition, the more time young people spent at home with their families, the less likely they were to gain access to these substances.

### Strengths and limitations

Strengths of our study include the use of longitudinal data collected over a period of up to 3 years prior to the pandemic and further assessments covering the two lockdowns, which allowed for a more comprehensive understanding of the impact of the pandemic. A clear strength is also the combination of data from the youth population as well as from patients with pre-existing mental illness, with both groups assessed under the same study protocol. This enabled the investigation of vulnerability and resilience and improved our understanding of how distinct groups of people may respond to challenging circumstances. However, some limitations should be acknowledged. First, our study had a relatively low response rate and high attrition during the data collection phase. It did not include underrepresented groups, such as participants from ethnic minorities that may have been disproportionately affected by the pandemic. Moreover, our clinical sample was relatively small, with the majority of participants being females. All of this may limit the generalisability of our findings. In addition, although our study used validated instruments (PHQ-9, AUDIT and EDE-Q) to measure psychiatric symptoms, these are not diagnostic tools but only measure a greater risk of the presence of clinical illness. Finally, psychiatric assessments were only conducted during periods of confinement, which precluded investigation of mental health changes once restrictions were lifted.

### Clinical implications

In summary, our study revealed opposite effects of the pandemic on mental health in youth with and without mental illness. Improvements in depression, alcohol misuse or eating disorder symptoms were observed over the course of the pandemic for participants with a higher pre-pandemic risk for these disorders, suggesting that the pandemic and lockdown measures decreased the mental health burden specifically in this population group. By contrast, the increases in depressive and eating disorder symptoms in those with low prior risk suggest the detrimental effects of such measures on healthier youth. If confirmed by future studies in a more representative sample, our findings could support personalised mental health interventions to help young people to cope better with the challenges of psychosocial stress and reduce the associated healthcare burden.

## Supporting information

Qi et al. supplementary material 1 Qi et al. supplementary material

Qi et al. supplementary material 2 Qi et al. supplementary material

## Data Availability

The data that support the findings of this study are available from the corresponding author (S.D.) on reasonable request.
