# Supplementary material for: Differing impact of the COVID-19 pandemic on youth mental health: combined population and clinical study
Source: BJPsych Open. 2023 Nov 20;9(6):e217. doi: 10.1192/bjo.2023.601 (PMC10753963; doi:10.1192/bjo.2023.601)
Supplement: Qi et al. supplementary material 1 — Qi et al. supplementary material [file S2056472423006014sup001.docx]

# Supplementary Materials

# 1. Supplemental Methods

# Study design

The pre-pandemic (pre-PD) data were obtained from 2016 to 2018 from the IMAGEN (follow-up 3; age 23), STRATIFY, and ESTRA studies, which used the same study protocol. Participants from these studies were invited to complete surveys (see below) online during the pandemic. The first survey was administered between 30^th^ April to 16^th^ May 2020 when the first national lockdown in the UK, France and Germany was imposed, respectively. It collected both pre-pandemic (3 months prior) data and data related to the first lockdown (LD1). From 16^th^ May to 4^th^ July 2020, a follow-up survey was administered when the first lockdown was released, during which time a shortened version of the assessments was sent to reduce the burden on participants. The third survey (follow-up 2) with the complete assessments was sent from 8^th^ to 26^th^ November 2020, when the second lockdown was imposed.

# Assessments

***The COVID-19 survey:*** This survey was adapted from The CoRonavIruS Health Impact Survey (CRISIS v0.1 <http://www.crisissurvey.org>) (1). The following measures were used in this study:

*Positive life changes*: questions in the 'life changes due to the pandemic in the last two weeks' that measured social interaction, family relationships, changes in living situations, and stressors related to those changes (questions 3 to 12 and 14) were used to compute a summary score. Reverse coding was used for questions 4, 6, 8, 10, 11, and 12. All questions were graded on a scale of 0 to 4. The total score ranged from 0 to 44, with a higher score indicating more positive life changes caused by the pandemic.

*Frequency of exercising*: This was measured by the question ‘during the three months prior to the onset of the COVID-19 crisis in your area/during the past two weeks how many days per week did you exercise (e.g., increased heart rate, breathing) for at least 30 minutes’ on a scale of 0 to 4. A higher score indicates a higher frequency of exercising.

*Media use frequency*: ‘media use during the past three months (baseline survey) or two weeks (follow-ups) included questions related to using digital media, social media and video games. A higher score indicates more time spent on media use.

*Daily food consumption*: This was measured by the question ‘during the past three months/two weeks, how much did you eat on average per day’ on a scale of 0 to 4. A higher score indicates a higher amount of food intake.

*Frequency of substance use*: The ‘substance use’ section of the COVID survey included 4 questions relating to substance use, either during the three months prior to the onset of the COVID-19 crisis or during the past two weeks. These questions measured the frequency of alcohol, cigarettes, marijuana, and other drugs (such as opiates, heroin, etc.) use on a scale from 0 to 4. The total score of substance use frequency ranged from 0 to 16, with a higher score indicating a higher frequency of substance use.

*Emotions/Worries*: These were assessed by summing up questions 2 through 12 in the ‘emotions/worries’ section, which measured the symptoms of anxiety and depression over a three-month/two-week period. Reverse coding was used for questions 3 and 4. All questions were graded on a scale of 0 to 4, with a higher score indicating higher worries. The total score of emotions & worries ranged from 0 to 44, with a higher score indicating higher worries related to COVID-19.

*COVID-related worries*: these were measured by the questions under ‘COVID-19 health/exposure status’ section, ‘during the past two weeks, how worried have you been about…’. Four sub-questions were involved, including worries about infection, family and friends being infected and potential impact on physical and mental health. All questions were graded on a scale of 0 to 4, with a higher score indicating higher worries.

# 2. Supplemental Results

# Behavioural trajectories during the pandemic

Mixed-effects ANOVAs were conducted to investigate the trajectories of changes in lifestyle and behaviours during the pandemic in the two cohorts. Significant main effects of time on positive lifestyle changes (F(2, 772) = 32.54, *p* < .001, η_p_^2^ = 0.078), frequency of media use (F(3,1146) = 27.11, *p* < .001, η_p_^2^ = 0.066), average daily eating (F(3,1152) = 4.45, *p* = .004, η_p_^2^ = 0.011) and frequency of substance use (F(3,1146) = 11.56, *p* < .001, η_p_^2^ = 0.029) (Supplemental Table 2, Supplemental Figure 1, A-E) were observed when analyzing the both samples together. Nominally significant effects of time (F(3,1152) = 2.70, *p* = .045, η_p_^2^ = 0.007) were found for frequency of exercising. Post-hoc analyses indicated significant increase in positive lifestyle changes after lockdown compared to during lockdowns (LD1 or LD2; *p* < .001), and increase in media use during the first lockdown, which decreased afterward but remained higher than pre-lockdown levels (all, *p* < .01). Conversely, daily food consumption specifically dropped after the first lockdown (pre-LD1 > post-LD1, *p* = .005; and LD1 > post-LD1, *p* = .01) (pre-LD1 > post-LD1, *p* = .005; and LD1 > post-LD1, *p* = .01); and substance use dropped during the lockdowns (pre-LD1 > LD1 and pre-LD1 > LD2, *p* < .001; post-LD1 > LD1 and post-LD1 > LD2, *p* < .05). Significant cohort effects revealed more positive lifestyle changes (F(1,385) = 27.31, *p* < .001, η_p_^2^ = 0.066) and eating (F(1,383) = 4.44, *p* = .04, η_p_^2^ = 0.015) in the population, and more media (F(1,381) = 6.13, *p* = .01, η_p_^2^ = 0.016) and substance (F(1,381) = 12.66, *p* < .001, η_p_^2^ = 0.032) use in the clinical sample. Males reported more positive lifestyle changes (F(1,385) = 6.74, *p* < .01, η_p_^2^ = 0.017), media use (F(1,381) = 11.43, *p* < .001), eating (F(1,383) = 5.80, *p* = .02) and substance use (F(1,381) = 7.20, *p* = .008, η_p_^2^ = 0.019) than females. No significant time x cohort or time x sex interactions were observed.

# Emotional trajectories during the pandemic

Similarly, we investigated emotions and worries, and COVID19-related worries (Supplemental Table 2, Figure 2A-E). There were significant main effects of time on anxiety symptoms, as assessed by the ‘emotions and worries’ section of the survey (F(3,1146) = 13.51, *p* < .001, η_p_^2^ = 0.034), worries about oneself being infected (F(2,780) = 13.96, *p* < .001, η_p_^2^ = 0.035), worries about friends or family being infected (F(2,780) = 56.31, *p* < .001, η_p_^2^ = 0.126) and worries about own physical (F(2,780) = 12.97, *p* < .001, η_p_^2^ = 0.032) and mental (F(2,780) = 28.84, *p* < .001, η_p_^2^ = 0.069) health. Unsurprisingly, emotions and worries were significantly higher throughout as compared to prior the pandemic (all, *p* < .001); worries about oneself and about own mental and physical health were higher during each lockdown compared to after lockdown (all, *p* < .001). A similar pattern occurred for worries about friends or family being infected, except that worries during the second lockdown remained significantly lower than during the first lockdown (all, *p* < .001). Patients and females worried significantly more on most measures (all, *p* < .05), except for worries about oneself being infected for which there was no significant cohort effect. There was no significant time x cohort or time x sex interactions for any outcome.

# 3. Group authorship

**IMAGEN Consortium**

Tobias Banaschewski, Gareth J. Barker, Arun L. W. Bokde, Uli Bromberg, Christian Büchel, Herta Flor, Antoine Grigis, Hugh Garavan, Penny Gowland, Andreas Heinz, Bernd Ittermann, Jean-Luc Martinot, Marie-Laure Paillère Martinot, Frauke Nees, Dimitri Papadopoulos Orfanos, Tomáš Paus, Luise Poustka, Juliane H. Fröhner, Michael N. Smolka, Henrik Walter, Robert Whelan, Sylvane Desrivières, Gunter Schumann

**ESTRA Consortium**

Marina Bobou, Zuo Zhang, Lauren Robinson, Gareth J. Barker, Arun L. W. Bokde18, Hervé Lemaître, Dimitri Papadopoulos Orfanos, Gunter Schumann, Ulrike Schmidt, Sylvane Desrivières

**STRATIFY Consortium**

Marina Bobou, M. John Broulidakis, Betteke Maria van Noort, Zuo Zhang, Lauren Robinson, Nilakshi Vaidya, Jeanne Winterer, Yuning Zhang, Sinead King, Gareth J. Barker, Arun L. W. Bokde, Hervé Lemaître, Frauke Nees, Dimitri Papadopoulos Orfanos, Julia Sinclair, Argyris Stringaris, Henrik Walter, Robert Whelan, Sylvane Desrivières, Gunter Schumann

# 4. Supplemental references

1. Nikolaidis A, Paksarian D, Alexander L, Derosa J, Dunn J, Nielson DM, et al. The Coronavirus Health and Impact Survey (CRISIS) reveals reproducible correlates of pandemic-related mood states across the Atlantic. Sci Rep. 2021; 11(1): 8139.
